# *Malassezia globosa* lipidome: The dynamics of uptake and secreted lipids

**DOI:** 10.1080/21505594.2026.2613494

**Published:** 2026-02-02

**Authors:** Catherine Eliana Cabrera Díaz, Mónica P. Cala, Elizabeth Jiménez-Díaz, Adriana Marcela Celis Ramírez

**Affiliations:** a Applied Biochemistry Research Group (GIBA), Department of Chemistry, Universidad de Los Andes, Bogotá, Colombia; b Metabolomics Core Facility (MetCore), Vicepresidency for Research, Universidad de Los Andes, Bogotá, Colombia; c Grupo de Investigación Celular Y Molecular de Microorganismos Patógenos (CeMoP), Department of Biological Sciences, Universidad de Los Andes, Bogotá, Colombia

**Keywords:** Lipidomic, *Malassezia*, lipid metabolism, lipid dynamics, secreted lipids, uptake lipids

## Abstract

*Malassezia globosa* plays a crucial role as part of the human skin’s mycobiome. However, this yeast has been detected in other niches, such as the gut. Despite being commensal, the pathogenic link in several dermatological conditions, but recently, chronic diseases such as cancer, Crohn’s disease, and Parkinson’s disease, among others, have been explored. Lipids can be involved in fungal pathogenesis, and this yeast is characterized by a significant lipid metabolic versatility, with a lack of the complex fatty acid synthase (FAS) required for the de novo synthesis of fatty acids, as it relies on lipase-releasing enzymes. Here, we assess lipid dynamics (lipids consumed vs. lipids secreted) using lipidomic analysis in the supernatant of mDixon media during two growth phases. 87 lipids within 17 classes of lipids were identified in three different lipid uptake-secretion patterns. Some lipids were characteristic, including the presence of glycochenodeoxycholic acid, glycerophospholipids (such as phosphocholine), cardiolipins, and sphingolipids (such as Cer-PI). Interestingly, sterols, bile acids, cholic acid and its derivates, some phosphocholines, fatty acyls, and cardiolipins were lipids consumed over time. The dynamic consumption of these lipids could presume an intriguing role in the metabolism of lipid processes in this yeast that could determine the interaction process and its pathogenic role.

## Introduction

*Malassezia* is a genus of lipophilic and lipid-dependent yeast, the principal constituent of the microbiota in human and animal skin. This lipid-dependent characteristic is attributed to the absence of fatty acid synthases, a multifunctional enzyme essential for the de novo synthesis of long-chain saturated fatty acids, such as palmitic acid [1]. In counterpart, *Malassezia* has a high number of genes encoding secreted hydrolases such as lipases, phospholipases, and sphingomyelinases, responsible for degrading sebum, a lipid-rich substance produced by the sebaceous glands [1–5].

The uptake of these resources is necessary for subsequent use in lipid-biosynthesis routes and is required to sustain the *Malassezia* species’ growth [1]. However, perturbation of these processes might relate to the establishment of some skin diseases, such as dandruff, atopic eczema, seborrheic dermatitis, pityriasis versicolor (PV), and folliculitis, and some systemic infections in immunocompromised patients [2,6–9]. Of the 21 species of *Malassezia* that have been described and divided into three clades up to now, clade B is related to the most common human skin resident, in which *M. globosa* is an important representative [10–12]. This species is strongly associated with skin conditions such as dandruff/seborrheic dermatitis and pityriasis versicolor, both of which are common superficial infections. Epidemiological reports estimate that dandruff and seborrheic dermatitis affect up to half of the world’s population [7,13].

Beyond dermatological disorders, emerging hypotheses suggest a potential role for *Malassezia* in chronic diseases, including colorectal cancer (CRC), inflammatory bowel disease (IBD), and various neurodegenerative disorders [14]. These associations raise important questions about the actual contribution of this yeast to the development of such pathological conditions [15]. Supporting this idea, *Malassezia* has also been detected in the gastrointestinal (GI) tract [16]. Recent perspectives further emphasize the systemic impact of *Malassezia*, describing its ability to traverse host niches from the skin and gut to the brain, where it may contribute to neurological consequences [17].

Lipids are key molecules important in intra- and inter-cellular signaling. These molecules are critical in cellular events such as cell growth, replication, differentiation, senescence, apoptosis, signal transduction, transcription, and stress responses [18]. In pathogenic fungi, lipids are signaling molecules that trigger and mediate specific cellular processes such as cell growth, proliferation, apoptosis, and senescence [19]. Recent Raman-based and lipidomic studies have confirmed variations among *Malassezia* species based on their lipid metabolic profiles [20,21]. In particular, noninvasive lipidomic profiling of oxylipin production has shown how *Malassezia* interacts with host eicosanoids, linking yeast lipid metabolism directly to human skin immune responses [22]. These studies suggest a differential utilization of lipid supplements among *Malassezia* spp., opening the door to explore the different roles of lipids in the *Malassezia* life cycle and their pathogenic role.

Specifically, pathogenic mechanisms and secretory processes in microbes are closely associated with the secretion of virulence factors for several prokaryotic and eukaryotic pathogens [18,23,24]. The secretion of proteins, lipids, and even polysaccharides, mainly via vesicle formation mechanisms, has been associated with pathogenicity in different yeasts, including *Cryptococcus neoformans*, *Histoplasma capsulatum*, and *Malassezia sympodialis* [24–28]. However, there is still much to know about the role of lipids in fungal virulence and life cycles.

The pathogenic role of *Malassezia* remains intriguing. Within the lipid metabolism of *Malassezia*, previous reports have shown that oleic acid, a representative fatty acyl (FA), can induce scalp flaking in susceptible individuals, supporting that *Malassezia* might be capable of FA-induced barrier disruption in patients with dandruff, possibly related to the absence of Δ9-desaturase leading to the incapability to catalyze the conversion of saturated to an unsaturated fatty acid [21,29]. Other virulence factors, such as azelaic acid, a dicarboxylic acid produced by *Malassezia furfur*, are associated with developing the PV phenotype [30]. Although the secretion of different hydrolases and some virulence factors has been described, there are only a few reports about the dynamics of the lipid composition of *M. globosa*.

The present study uses lipidomic analysis to investigate the consumed and secreted lipids in complex mDixon broth by *M. globosa*. Specifically, this article focuses on characterizing the in vitro lipid interaction based on a growth medium versus supernatant analysis of *M. globosa* in two different growth stages. These results will provide insight into the dynamics of this species regarding its lipid metabolism and help us hypothesize how these key components drive yeast metabolism and influence host-associated behavior, potentially biasing it toward commensalism or pathogenicity.

## Materials and methods

### Strains and culture conditions

The reference strain *Malassezia globosa* CBS 7966 (Westerdijk Institute, Utrecht, The Netherlands) was used. The strain was reactivated and maintained in modified Dixon (mDixon) agar [36 g L-1 mycosel agar [BD, USA], 20 g L-1 Ox Bile [Sigma Aldrich, USA], 36 g L-1 malt extract [Oxoid, UK], 0.02% glycerol [Sigma Aldrich, USA], 0.02% oleic acid [Sigma Aldrich, USA], and 0.1% Tween 40 [Sigma Aldrich, USA] [10].

### Experimental design

Two treatment groups were established: one group consisted of yeast growth at 72 h, and the other at 90 h. From both groups, *M. globosa* supernatant was analyzed. mDixon fresh broth was used as a control. To conduct the experiments, we follow the next steps: 1. The strain was grown on mDixon agar at 33 °C for 5 d. 2. One colony was suspended in 3 mL of inoculum in water plus 0.1% Tween 80 [Sigma Aldrich, USA] and adjusted to McFarland standard 2. 3. Then, this inoculum was used to inoculate 27 mL of mDixon broth, 36 g L-1 malt extract [Oxoid, UK], 6 g L-1 peptone [BD, USA], 20 g L-1 Ox bile [Sigma Aldrich, USA], 0.02% glycerol [Sigma Aldrich, USA], 0.02% oleic acid [Sigma Aldrich, USA], and 0.1% Tween 40 [Sigma Aldrich, USA] for 96 h at 33°C and 180 rpm. 4. An aliquot of 300 µL was used to inoculate 12 flasks, each containing 29.7 mL of fresh mDixon broth. Six flasks corresponding to independent biological replicates were incubated for 72 h and six for 90 h, to the early stationary and stationary phases, respectively. From each biological replicate, three technical replicates were analyzed to ensure reproducibility. All cultures were incubated at 33 °C with shaking at 180 rpm [31,32].

#### Lipid extraction

Treatment groups were centrifuged at 4500 rpm for 10 min, and supernatants were collected. Then, 5 mL of isopropanol was added to the supernatant and centrifuged at 4500 rpm for 10 min. Lipid extraction was performed according to the classical Bligh and Dyer method [33], with some modifications [34–36]. 2 mL of a citric acid buffer [0.1 M sodium citrate tribasic dihydrate, 1 M sodium chloride, pH 3.6], 2 mL of MeOH, and 4 mL of chloroform were added to 8 mL of supernatant collected previously. The mixture was homogenized with a vortex for 15 min and sonicated for 30 min. The extracted lipids’ organic phase was collected and dried on a Speed Vac. Then, the dry extract was re-dissolved in 1 mL of ACN containing 0.1% NH_3_·H_2_O (v/v), followed by strong anion-exchange solid-phase extraction using Strata SAX SPE-cartridge (55µM, 70 A, 100 mg, 1 mL Phenomenex) which was pre-conditioned with 3 mL ACN. After sampling 1 mL of the lipid extract, the cartridge was washed with 3 mL acetone/H2O (1/9, v/v), 3 mL acetone, and eluted with 3 mL formic acid/acetone (1/99, v/v) followed by evaporation using a Speed Vac. Samples were stored at −86°C for 1 week and dissolved in 1 mL of MeOH for further analysis [34–36].

#### Lipidomic analysis

Lipid analysis was performed by the MetCore at Universidad de los Andes (Bogotá, Colombia) (https://metcore.uniandes.edu.co/es/) using Agilent Technologies 1260 LC coupled with a 6545 Q-TOF quadrupole time-of-flight mass analyzer with electrospray ionization. A dilution of 1:100 was necessary before the injection. 1 µL of each sample was injected into an InfinityLab Poroshell 120 EC-C18 column (100 ×3.0 mm, 2.7 µm). Chromatographic analysis was carried out at 65°C and constant flow 0.6 mL/min using gradient elution with phase A (60:40 ACN: type I water with 10mM of ammonium formate and 0.1% v/v of formic acid) and mobile phase B (90:10 Isopropanol: Acetonitrile with 10mM of ammonium formate and 0.1% v/v of formic acid). The elution gradient was 0 min 15% (B), 0–4 min 30% (B), 4–5 min 48% (B), 5–22 min 82% (B), 22–23 min 95% (B), 23–25 min 95% (B), 25–26 min 15% (B), and 26–31 min 15% (B). Mass spectrometry detection was performed in positive and negative ionization mode in a full scan from 100 *m/z* to 1200 m/z. The mass correction was performed during the analysis with reference masses *m/z* 121.0509 (C_5_H_4_N_4_) y m/z 922.0098 (C_18_H_18_O_6_N_3_P_3_F_24_).

A tentative identification based on the *m/z* of the compounds showing statistically significant differences was searched against several online databases using the search engine CEU MassMediator (http://ceumass.eps.uspceu.es/mediator) [37]. Molecular features assigned to lipids from the databases were based on mass accuracy (maximum error mass of 10 ppm). Molecular features were also inspected with the Agilent MassHunter Qualitative Analysis Software B.10.00. MS/MS spectra and the Lipid annotator library were used to identify the lipid compounds [38,39]. A unique ID was given to each lipid based on its retention time, mass–charge ratio, molecular formula, monoisotopic distribution, adduct formation, and MS/MS spectra. Lipid classification was done based on lipid maps categorization (https://www.lipidmaps.org/databases/lmsd/browse) [40]. All data regarding lipidomic analysis have been submitted to the Metabolomics Workbench (PR002156), which can be accessed at: http://dx.doi.org/10.21228/M8XR64 [41].

### Statistical analysis

Univariate (UVDA) and Multivariate (MVDA) data statistical analyses were performed to investigate differences among the molecular features detected in the two treatment groups and the control.

For UVDA, the normality of data was evaluated with the Shapiro–Wilk test (*p* ≤ 0.05). Wilcoxon test (*p* ≤ 0.05) was performed on all the possible pairings to assess differences for an individual metabolite in each pair. Additionally, differences among all the groups were evaluated by performing a non-parametrical Kruskal–Wallis test (*p* ≤ 0.05). Posthoc, pair-wise analysis was performed using the Dunn test to conclude which group the metabolite presents a significant difference. Finally, the false discovery rate at level α = 0.05 for all UVDA analyses was controlled by the Benjamini−Hochberg correction test. UVDA analysis was performed using R version 4.0.0 (https://www.rstudio.com/) [42].

For MVDA, unsupervised (PCA, principal component analysis) and supervised (PLS-DA partial least-squares discriminant analysis and OPLS-DA, orthogonal PLS-DA) (Supplementary Figures S2-S6) models were performed to determine differences in molecular feature intensity between groups. All models were evaluated by the explained variance (R2) and the predicted variance (Q2) quality parameters. The Variable Importance Parameter (VIP) ≥1 and jackknifing confidence interval not including zero were also selected as statistically significant from the OPLS-DA models. All the analyses were performed in SIMCA 14.1. A heat-map graphical representation of the differential lipid expression using the relative intensities obtained from the detected lipids was performed using the heatmap package in R version 4.0.0 (https://www.rstudio.com/) [42].

## Results

### Lipid differentiation of *Malassezia* mDixon supernatant at different growth hours.

**Figure 1.**
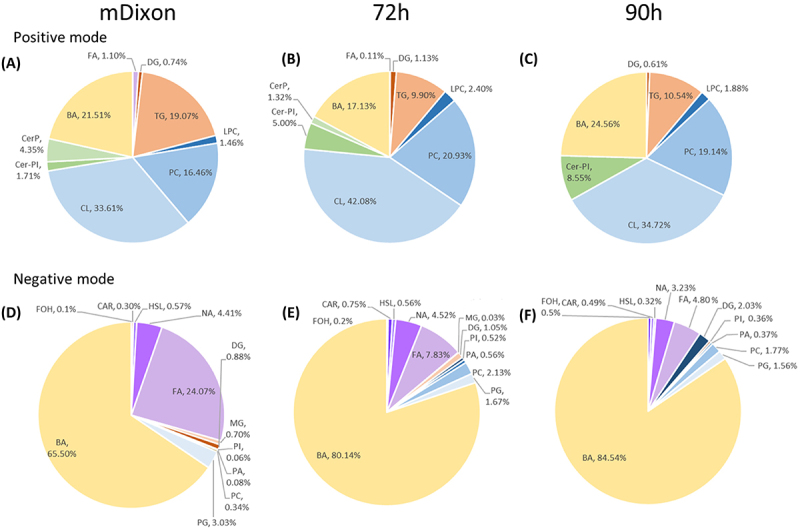
Relative percentage of differential lipid classes in *Malassezia globose* detected in positive mode at (A) mDixon (control) (B) 72 h of growth and (C) 90 h of growth, and in negative mode at (D) mDixon (control) (E) 72 h of growth and (F) 90 h of growth. Lipid classes are grouped by lipid families, represented in different colors. Fatty acyls (FA): purple, Glycerolipids (GL): orange, glycerophospholipids (GP): blue, Sphingolipids (SP): green, sterol lipids (ST): yellow. The following acronyms were used to identify the different lipid classes: fatty acids and Conjugates (FA), fatty acylcarnitines (CAR), fatty alcohols (FOH), fatty acyl homoserine lactones (HSL), fatty amides (NA), triacylglycerols (TG), Diacylglycerols (DG), Monoacylglycerols (MG), Cardiolipins (CL), Diacylglycerophosphates (PA), Diacyglycerophosphocholines (PC), Glycerophosphoglycerols (PG), Glycerophosphoinositols (PI), Monoacyglycerophosphocholines (LPC), ceramide phosphates (CerP), Phosphosphingolipids (Cer-PI) and bile acids (BA).

Five lipid families were identified in both positive and negative ionization modes (Tables 1 and 2): Fatty acyls (FA), Glycerolipids (GL), Glycerophospholipids (GP), Sphingolipids (SP), and sterol lipids (ST). From there, 17 lipid classes were identified: Fatty Acids and Conjugates (FC), Fatty acylcarnitines (CAR), Fatty alcohols (FOH), Fatty acyl homoserine lactones (HSL), Fatty amides (NA), Triacylglycerols (TG), Diacylglycerols (DG), Monoacylglycerols (MG), Cardiolipins (CLs), Diacylglycerophosphates (PA), Diacyglycerophosphocholines (PC), Glycerophosphoglycerols (PG), Glycerophosphoinositols (PI), Monoacyglycerophosphocholines (LPC), Ceramide (Cer), Phosphosphingolipids (Cer-PI), and Bile acids (BA) (Figure 1).

**Table 1.** Relative abundance of 52 differentially present lipids in fresh mDixon control media and the supernatant at 72 h and 90 h of growth. Analysis was carried out in positive ionization mode.

Lipid families and lipid classes	Lipid quantity in fresh mDixon control (%)	Lipid quantity in supernatant 72 h (%)	Lipid quantity in supernatant 90 h (%)
**Fatty acyls (FA)**			
Fatty Acyls (FA)	1.098 ± 0.085	0.114 ± 0.039	0 ± 0
**Glycerolipids (GL)**			
Diacylglycerols (DG)	0.74 ± 0.051	1.125 ± 0.372	0.612 ± 0.087
Triacylglycerols (TG)	19.071 ± 0.754	9.896 ± 2.243	10.536 ± 1.486
**Glycerophospholipids (GP)**			
Cardiolipins (CL)	33.605 ± 0.117	42.081 ± 0.668	34.723 ± 0.284
Monoacyglycerophosphocholines (LPC)	1.458 ± 0.036	2.399 ± 0.354	1.879 ± 0.195
Diacyglycerophosphocholines (PC)	16.458 ± 0.079	20.932 ± 0.338	19.138 ± 0.221
**Sphingolipids (SP)**			
Ceramides phosphates (CerP)	4.354 ± 0.205	1.32 ± 0.343	0 ± 0
Phosphosphingolipids (Cer-PI)	1.708 ± 0.07	5.003 ± 1.374	8.551 ± 0.806
**Sterol Lipids (ST)**			
Bile acids (BA)	21.507 ± 0.112	17.131 ± 0.232	24.562 ± 0.287

**Table 2.** Relative abundance of 37 differentially present lipids in fresh mDixon control media and the supernatant at 72 h and 90 h of growth. Analysis was carried out in the negative ionization mode.

Lipid families and lipid classes	Lipid quantity in mDixon control (%)	Lipid quantity in 72 h (%)	Lipid quantity in 90 h (%)
**Fatty acyls (FA)**			
Fatty acylcarnitines (CAR)	0.299 ± 0.025	0.747 ± 0.092	0.49 ± 0.073
Fatty Acyls and Conjugates (FC)	0.073 ± 0.013	0.24 ± 0.034	0.537 ± 0.039
Fatty alcohol (FOH)	0.566 ± 0.071	0.56 ± 0.077	0.316 ± 0.035
Fatty acyl homoserine lactones (HSL)	4.408 ± 0.133	4.523 ± 0.254	3.234 ± 0.146
Fatty amides (NA)	24.066 ± 0.196	7.826 ± 0.316	4.801 ± 0.109
**Glycerolipids (GL)**			
Diacylglycerols (DG)	0.879 ± 0.026	1.052 ± 0.094	2.032 ± 0.105
Monoacylglycerols (MG)	0.703 ± 0.055	0.035 ± 0.012	0 ± 0
**Glycerophospholipids (GP)**			
Diacylglycerophosphates (PA)	0.076 ± 0.007	0.562 ± 0.153	0.368 ± 0.044
Diacyglycerophosphocholines (PC)	0.337 ± 0.031	2.13 ± 0.312	1.766 ± 0.291
Glycerophosphoglycerols (PG)	3.031 ± 0.04	1.67 ± 0.195	1.561 ± 0.141
Glycerophosphoinositols (PI)	0.058 ± 0.006	0.517 ± 0.095	0.355 ± 0.032
**Sterol Lipids (ST)**			
Bile acids (BA)	65.504 ± 0.352	80.138 ± 0.764	84.538 ± 0.599

Using high-performance liquid chromatography combined with electrospray ionization mass spectrometry (HPLC/ESI-MS), a total of 968 molecular features were identified in the present study. Using these features as lipid profiles, a reduction of dimensionality via multivariate data analysis (MVDA) was performed to determine the relevant compounds across the three treatment groups. Eighty-five lipids of five lipid families and 17 lipid classes were identified through this analysis (Table S1 Supplementary Table S1, Table S2 Supplementary Table S2, Table S3 Supplementary Table S3) [43,44]. Complete lipid characterization of fresh mDixon media was not performed, as this work aimed to identify and correlate the lipid composition of consumed and secreted lipids at two stages of growth, 72 h and 90 h, compared to fresh mDixon media. A two-dimensional PCA analysis revealed differentiation among treatment groups and the control group (Supplementary Figure S1). The study confirmed excellent analytical performance throughout the reproducibility in QC‘s, and reproducibility among analytical replicates was also observed [43] (Supplementary Figure S1).

The supernatant of yeast cell cultures was analyzed during the early and late stationary phases (72 h and 90 h, respectively) in both positive and negative ionization modes. During the progression of the stationary phase, the analysis showed a different distribution of lipids; however, all five families (ST), (GP), (GL), (SP), and (FA) were predominant in both analyses. We observed a decrease in the relative percentage when comparing fresh medium to 72 h and 90 h supernatant of most families identified, except for (ST) and (Cer-PI) (Figure 1a-d compared to 1b-e and 1c-f), whose percentages increase throughout the growth of the yeast in mDixon media. Lipids with even and odd chains were detected. In some cases, sterols with the same chemical formula were assumed to be different compounds as retention time differs, and some bile acids have an identical mass (data not shown) and molecular formula to oxidized sterols lacking a carboxylic acid group [40]. Further analysis should clarify the nature and diversity of the sterols present. Levels of (PC) and (TG) remain unchanged throughout the stationary phase. In addition, a change in the length and unsaturation of FA was not observed, as the temperature remained constant throughout the experiment.

**Figure 2.**
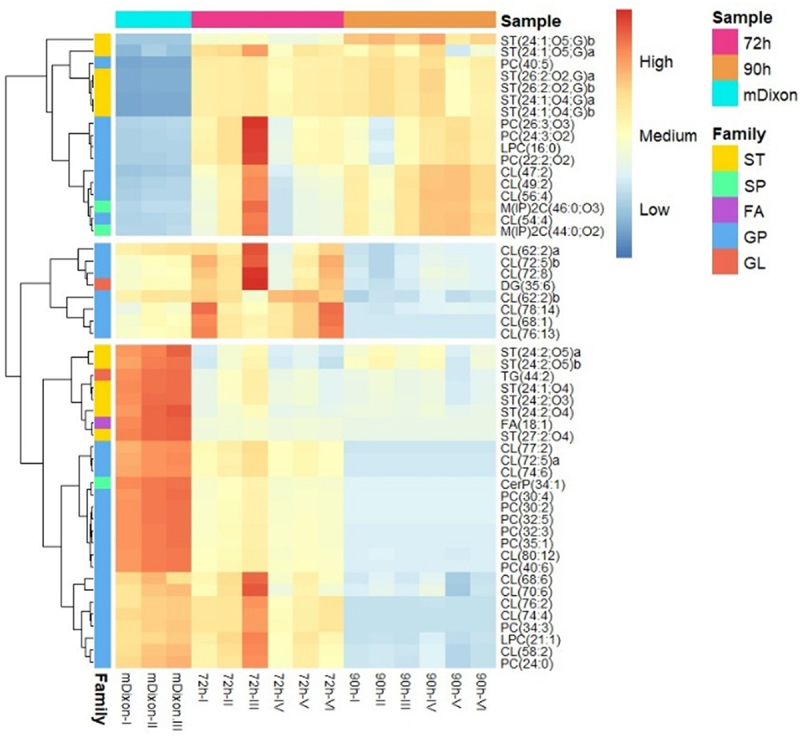
Heat map of identified molecular features in positive mode for differentiation of fresh mDixon (control) and supernatant of 72 h and 90 h of *M. globosa* growth. The heat map denotes each molecular feature’s higher (red) to lower (blue) relative intensity. Hierarchical divisions based on clustering are displayed, showing three possible different intake and secretion patterns. Fatty acyls (FA): purple, Glycerolipids (GL): orange, glycerophospholipids (GP): blue, Sphingolipids (SP): green, sterol lipids and (ST): yellow. The following acronyms were used to identify the different lipid classes: fatty acids (FA), triacylglycerols (TG), Diacylglycerols (DG), Cardiolipins (CL), Diacyglycerophosphocholines (PC), Monoacyglycerophosphocholines (LPC), ceramide phosphate (CerP), Phosphosphingolipids (Cer-PI) and sterols (ST).

**Figure 3.**
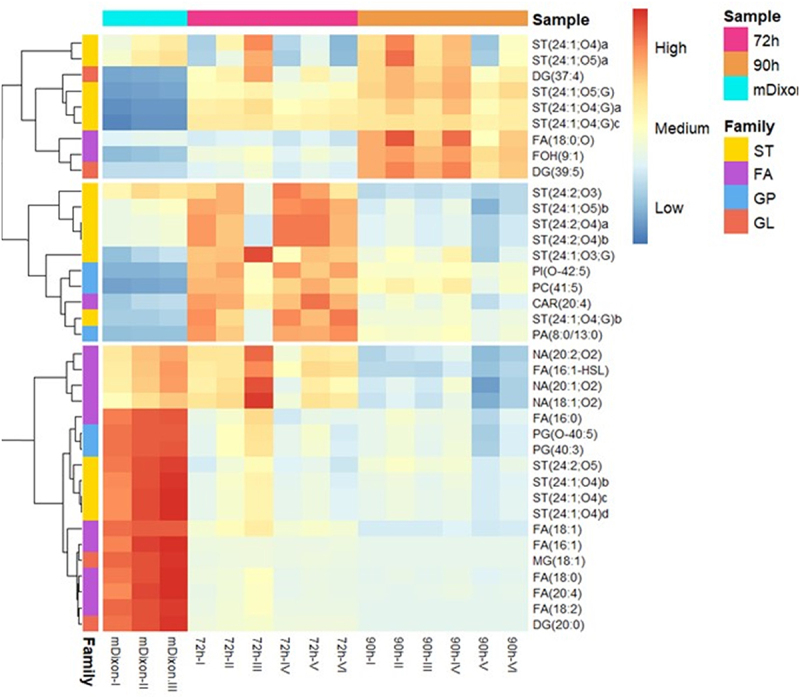
Heat map of identified molecular features in negative ionization mode for differentiation of the fresh mDixon (control) and supernatant of 72 h and 90 h of *M. globosa* growth. The heat map denotes each molecular feature’s higher (red) to lower (blue) relative intensity. Hierarchical divisions based on clustering are displayed, showing three possible different uptake and secretion patterns. Fatty acyls (FA): purple, Glycerolipids (GL): orange, glycerophospholipids (GP): blue and sterol lipids (ST): yellow. The following acronyms were used to identify the different lipid classes: fatty acyls and Conjugates (FA), fatty acylcarnitines (CAR), fatty alcohols (FOH), fatty acyl homoserine lactones (HSL), fatty amides (NA), Diacylglycerols (DG), Monoacylglycerols (MG), Diacylglycerophosphates (PA), Diacyglycerophosphocholines (PC), Glycerophosphoglycerols (PG), Glycerophosphoinositols (PI) and sterols (ST).

Heatmaps were constructed and analyzed in both positive and negative electrospray ionization modes to determine the changes in individual lipids within the treatment groups. Based on these results, three hierarchical divisions, based on clustering, are displayed for the positive ionization mode (Figure 2) and the negative ionization mode (Figure 3), respectively. Three different lipid uptake-secretion patterns were proposed for *M. globosa* based on these two figures; the first pattern was assigned to secreted lipids in the supernatant (Figure 2 and 3 top rectangles), the second pattern was assigned to the late uptake lipids, lipids whose amount only decreased at late stationary phase ~90 h (Figure 2 and 3, middle rectangle) and the final pattern was assigned to consumed lipids (Figure 2 and 3, bottom rectangle). From the families identified, glycolic acid derivates (Cer-PI), (oxPC), (FC), (FOH), and (TG) were observed in the first pattern corresponding to secreted lipids into the supernatant. PI’s, CAR, some other sterols, and CL were observed and associated with the second pattern corresponding to the lipids that were consumed or degraded late in the stationary phase by *M. globosa*, and finally, Cholesterol derivates, (FA), NA, HSL, MG, CL, PC, PG, and LPC were observed and assigned to the third pattern consumed lipids throughout the stationary phase.

## Discussion

The lipidomic analysis conducted here highlights the complexity of lipid metabolism in *M. globosa*. This yeast can colonize skin but also more complex human niches, including the gastrointestinal tract. The lipid composition found in the supernatants corresponds with the composition of mDixon. This growth medium is particularly suitable for studying lipid consumption and secretion. It contains a wide variety of lipids naturally encountered by microorganisms, including oleic acid, Tween 40, glycerol, and ox bile lipids, along with maltose and peptone [45–47]. The diversity of lipids such as linoleic, oleic, and palmitic acid, although not fully characterized in malt extract, supports a broad range of metabolic activities [48,49]. Most of the discriminatory variables detected and identified in this study are standard components of mammalian bile, including various bile acids and phospholipids, which underscores the relevance of the mDixon medium [46,50]. The finding of diverse glycinate bile acids was expected as they are conjugated with taurine or glycine for secretion into bile in metabolism [51]. A biochemical synthesis in the bile generates some of the identified acylglycerols in mDixon [52,53].

The presence of secondary bile acids, whose influence on the immune responses to pathogens is key, further emphasizes the medium’s suitability to study lipid interactions in a pathological context. Their role in fungal infections, specifically in modulating fungal presence, such as in *Candida albicans* through specific bile acids like taurocholic acid, could provide insights into yeast behavior and host–pathogen interactions [54,55]. Future studies are needed to explore alternative growth media, such as the artificial sebum-containing Leeming and Notman agar medium, to expand our understanding of lipidomic influences on *Malassezia* and other microorganisms [56].

Most of the families identified in this study have been previously described in *M. globosa* [21] (Celis Ramírez et al., 2020), except for the lipid ceramide phosphate (Cer-P) and phosphoinositol ceramides (Cer-PI) (Figure 1). These lipids play distinct but complementary roles in *M. globosa* and other pathogenic yeasts. Cer-P functions primarily as a bioactive sphingolipid involved in stress signaling, regulation of cell growth, and survival under adverse conditions, roles that parallel its function as a signaling lipid in higher eukaryotes [57]. Cer-PI, by contrast, represents the predominant sphingolipid species in fungi and serves as the functional equivalent of sphingomyelin in mammals. It contributes to plasma membrane integrity, polarity, and raft domain formation, which are essential for fungal growth and host interaction [58]. In pathogenic yeasts such as *C. albicans* and *C. neoformans*, Cer-PI and its mannosylated derivatives are further implicated in immune evasion and cell wall stability [59].

In contrast, in *Malassezia*, Cer-PI has been associated with extracellular vesicles that modulate host inflammatory responses in the skin environment [60]. Importantly, the enzyme IPC synthase, responsible for Cer-PI biosynthesis, is absent in mammals but essential in fungi. Furthermore, phosphoinositol ceramides may play a pivotal role in supporting yeast survival by modulating cellular differentiation and proliferation [59].

### Progressively consumed lipids

**Figure 4.**
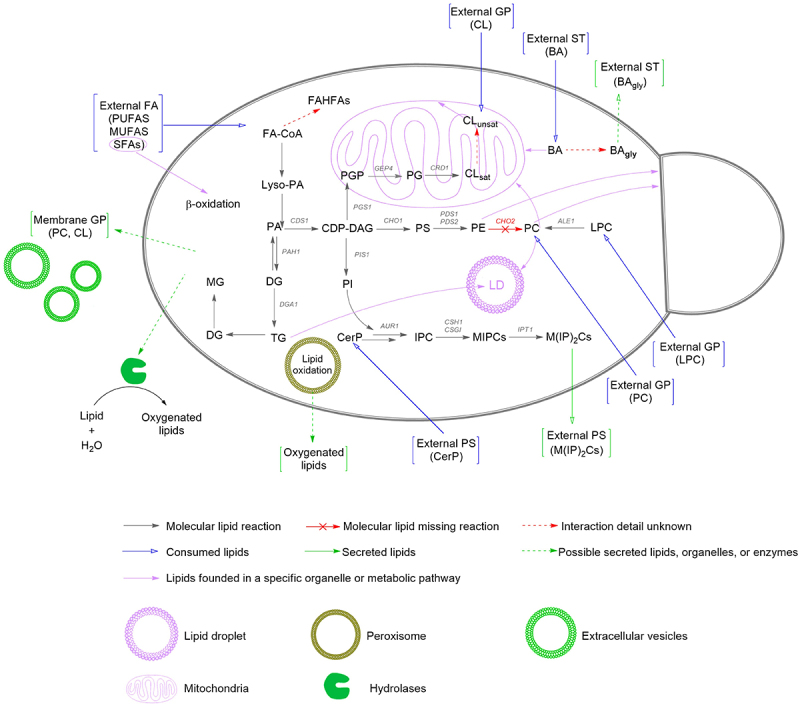
A general overview of consumed, secreted, and interactions of lipid metabolism in *M. globosa* based on lipidomic analysis and Figures 1, 2, and 3. Black arrows correspond to known lipid reactions; blue arrows correspond to consumed lipids in this study.

The observed changes in lipid profiles suggest a dynamic interaction involving either lipid consumption and secretion or lipid consumption and degradation in mDixon medium, with a higher proportion of lipids appearing to be consumed. These findings are consistent with previous reports on the lipid-dependent metabolism of *M. globosa* [20,21]. The main lipid classes consumed included carnitines, fatty acyls, glycerophospholipids, and bile acids (Figure 4). In particular, the consumption of cardiolipins (CL) and glycerophospholipids (GP) may be linked to their essential roles in the electron transport chain, stabilization of mitochondrial DNA in yeast, lipid droplet formation and stability, and overall cell maintenance [61–64]. Although all enzymes required for the de *novo* synthesis of CL are present in this species, the enzymatic machinery responsible for producing unsaturated CL remains unclear [21]. In *S. cerevisiae*, and to a lesser extent in other yeasts such as *C. albicans*, unsaturated fatty acids play a critical role in stabilizing cardiolipin and maintaining mitochondrial membrane integrity. Cardiolipin acyl-chain unsaturation is essential for proper mitochondrial respiration, cristae organization, and stabilization of respiratory chain super complexes [61,65,66]. In *S. cerevisiae*, defects in fatty acid desaturation or cardiolipin remodeling lead to compromised mitochondrial function and reduced stress tolerance [65,67]. Interestingly, in this study, several unsaturated cardiolipins were found to be consumed, suggesting that these lipids are at least partially acquired from the growth medium rather than synthesized de *novo*.

The absence of enzymes for the formation of PC, such as *CHO2* (phosphatidylethanolamine N-methyltransferase) or the Kennedy pathway enzymes, could explain the high CL consumption throughout the experiment [21]. Additionally, the presence of the *ALE1* enzyme (Lysophospholipid acyltransferase) suggests that the small quantity of LPC consumed could be converted to PC, as it plays a critical role in regulating the physical properties of membranes [68,69]. Other phospholipids, such as PE, a significant membrane component, could be formed through the CDP-DAG pathway [68,70], explaining the absence of consumption of these types of lipids in this study [68,70].

The consumption of bile acids, such as cholic acid and its derivatives, was observed (Figure 1). The role of these acids has been linked to the alteration of age-related processes in *S. cerevisiae*, increasing its lifespan through variations in the mitochondrial membrane lipidome. Therefore, consuming these external acids may help maintain cell function and serve as a carbon source for the yeast [62,71]. In addition, bile acids have been associated with fungistatic activity in other yeasts, such as *C. albicans*, suggesting that their effects vary across different fungal species [72,73]. In the case of *M. globosa*, the role of bile acids remains unexplored; however, the present study indicates that this yeast can utilize bile acids as a potential nutrient source. Moreover, bile acids may promote *Malassezia* persistence in the gut by enhancing its adhesion to mammalian cells, a phenomenon previously demonstrated for *C. albicans*. This observation suggests a possible mechanism linking *Malassezia* to gut-associated diseases, such as inflammatory bowel disease (IBD) and colorectal cancer (CRC) [54,55,74]. Regarding other sterols, cholesterol and closely related compounds have been reported in *M. globosa* and are also present in bile; however, cholesterol consumption was not detected in this study, suggesting that sterols may instead be synthesized endogenously [21].

For FAs, a wide variety, including PUFAs, MUFAs, and saturated acids, was consumed during the stages of this study using mDixon media. Its consumption was expected, as had been previously reported in *M. globosa* [21]. Considering the absence of fatty acid synthase and, differing from other *Malassezia* species, the lack of *OLE1* (FA Δ9-desaturase) in *M. globosa* explains the consumption of both long-chain saturated fatty acids, palmitic and oleic acids, which are essential for fatty acid metabolism, as they serve as building blocks for the synthesis of membrane lipids and storage lipids [75]. Additionally, saturated fatty acids play a pivotal role in the β-oxidation process, as *Malassezia* lacks the enzyme ECI1 (Δ3,2-enoyl-CoA isomerase) required for the oxidation of unsaturated fatty acids [21,76]. Additionally, in *M. globosa*, the consumption of FA could explain the synthesis of FAHFAS within the yeast and the presence of lipid droplets to promote lipid particle formation [21,64,77].

Consistent with these observations, sterols (ST) and specific fatty acids (FA) were also steadily consumed across all growth phases, reflecting their essential roles in maintaining membrane integrity and signaling. Sterols play a central role in the fungal membrane architecture, influencing fluidity, permeability, and raft-dependent signaling [78,79]. In *Malassezia*, sterol uptake is particularly critical given the absence of de novo fatty acid synthesis and reliance on exogenous lipid sources [1]. Continuous sterol consumption indicates a sustained requirement for membrane biogenesis and signaling during growth. Similarly, fatty acids are indispensable for energy production, phospholipid biosynthesis, and modulation of host–pathogen interactions [19]. The absence of detectable remodeling in FA chain length or unsaturation under constant temperature suggests that *M. globosa* maintains a stable lipid composition under these culture conditions, consistent with reports that FA remodeling is often triggered by temperature or oxidative stress [80]. In contrast to these continuously depleted species, a second group of lipids remained stable until the late stationary phase, when their levels began to decline.

### Late-uptake lipids

PC and TG showed a distinct “late-uptake” pattern, remaining at stable levels throughout the exponential and early stationary phase before declining at ~90 h. This delay suggests that these lipids are strategically preserved until external lipid sources become limiting. PC is the dominant bilayer-forming phospholipid in fungi and plays essential roles in membrane integrity, fluidity, and viability [78,81]. The stability of PC during most of the growth curve may reflect a safeguard mechanism, ensuring that *M. globosa* retains critical structural components until the late stationary phase, when nutrient stress intensifies.

TG, by contrast, functions as a neutral storage lipid, sequestering fatty acids within lipid droplets and preventing lipotoxicity [82,83]. Their conservation levels until the late phase suggest that *M. globosa* reserves TG as an emergency carbon and energy source, mobilizing only those when exogenous supplies are depleted. Such delayed turnover of neutral lipids parallels survival strategies in other fungi, where TG reserves are critical during nutrient limitation and environmental stress [84].

Levels of phosphatidylcholine (PC) and triacylglycerols (TG) remained unchanged throughout the stationary phase, suggesting that these lipid families are tightly regulated in *Malassezia*. PC is one of the dominant membrane phospholipids in fungi and plays a central role in maintaining bilayer stability, intrinsic curvature, and membrane fluidity [68,85]. In pathogenic yeasts, membrane phospholipid composition has also been shown to influence cell-wall organization and the exposure of immunogenic components such as β-glucans, thereby contributing indirectly to immune evasion [86,87].

In contrast, TG represents the major neutral lipid storage pool and functions as a reservoir of fatty acids that can be mobilized under nutrient limitation. In pathogenic yeasts, TG metabolism has been linked to morphogenesis, stress resistance, and survival in lipid-rich host environments [19,88]. In *Malassezia*, TG dynamics are closely associated with extracellular lipase activity, which enables hydrolysis of host sebum triacylglycerols and subsequent uptake of liberated fatty acids [89]. The stability of PC and TG observed here, therefore, likely reflects an adaptive strategy to preserve essential membrane architecture while maintaining access to endogenous lipid reserves during prolonged stationary-phase growth.

In general, the late decline of PC and TG may reflect a phase-specific metabolic prioritization in *M. globosa*, whereby exogenous lipids are utilized first, while endogenous structural and storage lipids are spared until external sources are exhausted. An additional possibility is that the preservation of these lipids supports stress adaptation or host interaction functions, consistent with the involvement of PC remodeling and TG metabolism in fungal pathogenesis in *Cryptococcus* and *Candida* [15,78,82]. This delayed depletion suggests that *M. globosa* focuses on the utilization of these lipids differently from those consumed throughout growth. Another distinct group showed the opposite trend, being secreted into the medium rather than consumed.

### Secreted lipids

Some molecules increased in the medium at 72 h but decreased later in the stationary phase. This pattern could reflect late uptake of these lipids, or alternatively, early uptake followed by later secretion after enzymatic degradation. Enzymes such as lipases, phospholipases, sphingomyelinases, and lipoxygenases are likely responsible for these processes in *Malassezia* metabolism [1–4,24,25]. Some lipids that followed this pattern were glycolic acids; these lipids could be associated with a possible toxicity effect within the cell, as was observed in other yeasts [72,73]. Oxygenated lipids are more likely to be the product of hydrolase secretion of *Malassezia* but also could be generated as a response of the cell to stress, as the accumulation of oxidized lipids is a result of an increase of peroxisomes and can be associated with danger signals; as they can generate oxidative stress and cell death, therefore its secretion may help in the regulation of their harmful effect inside the cell [80,90,91]. Therefore, oxygenated lipids could be associated with *M. globosa* pathogenic role [18,89,91–93]. Lipid secretion is increasingly recognized as an important feature of pathogenic fungi, often mediated by extracellular vesicles (EV), which deliver lipids, proteins, and other virulence factors to the host environment [27]. Additionally, the secretion of Cer-PI, ST, and related sphingolipids have been implicated in fungal cell wall integrity and immune evasion [18,58]. Moreover, oxidized PC and fatty alcohols may represent metabolic byproducts with immunomodulatory potential. Importantly, secreted oxylipins derived from lipid metabolism have been linked to modulation of host eicosanoid pathways, suggesting a direct role in skin inflammation [22]. Thus, the secretion of structurally diverse lipids by *M. globosa* underscores their dual role as metabolic end-products and putative virulence factors. The possible secretion of some membrane lipids, such as (PC) and (CL) could be associated with extracellular vesicle secretion, as reported for other yeast, such as *M. furfur* or *C. neoformans* [24,93,94].

Together, these three patterns reveal a coordinated strategy in *M. globosa* lipid metabolism. The yeast balances secretion, preservation, and consumption depending on the growth phase. Lipid secretion, particularly of Cer-PI and oxPC, may serve pathogenic functions by modulating host responses, while the late mobilization of PC and TG highlights metabolic prioritization under nutrient limitations. The continuous uptake of some sterols and fatty acids underscores their essential roles in membrane maintenance and signaling. Beyond skin-associated commensalism, such metabolic adaptations may enable *Malassezia* to persist in diverse host niches, consistent with emerging evidence linking this genus to gastrointestinal and even neurological disorders [15]. Overall, in *Malassezia*, secreted lipids could serve multiple functions: (i) modulation of host lipid metabolism, (ii) interference with skin barrier integrity, and (iii) signaling roles in host–pathogen communication.

In conclusion, *M. globosa* exhibits a dynamic and complex lipid metabolism that is highly dependent on external lipids, with distinct consumption and secretion patterns linked to nutrient acquisition, stress responses, and potential pathogenicity. The detection of ceramide species (ceramide phosphate, Cer-P, and phosphoinositol ceramides, Cer-PI) together with the involvement of bile acids highlights novel avenues for exploring host–yeast interactions. These findings emphasize the importance of conducting *in vivo* studies using both invertebrate (e.g. *Galleria mellonella*) and vertebrate (e.g. murine) models to validate lipid-associated mechanisms. Furthermore, integrating multi-omics approaches with advanced technologies, such as single-cell transcriptomics, will be essential for unraveling the metabolic networks of this yeast in greater depth and for more precisely defining its role in health and disease. Finally, extending these investigations to other relevant species, such as *M. restricta*, will provide a more comprehensive understanding of the complex metabolism of key constituents within the human microbiota.

## Supplementary Material

Supplemental Material

Supplemental Material

Supplemental Material

Supplemental Material

Supplemental Material

Supplemental Material

Supplementary Information.docx

Supplementary Table 1.docx

Supplementary Table 2.xlsx

Supplementary Table 3.xlsx

## Data Availability

The authors confirm that the data supporting the findings of this study are available within the article and its supplementary materials. The data that support the findings of this study are openly available in the Metabolomics Workbench at http://dx.doi.org/10.21228/M8XR64, reference number (PR002156)[41].
