# Supplementary figures and images for: Malassezia globosa lipidome: The dynamics of uptake and secreted lipids
Source: Virulence. 2026 Feb 2;17(1):2613494. doi: 10.1080/21505594.2026.2613494 (PMC12944816; doi:10.1080/21505594.2026.2613494)

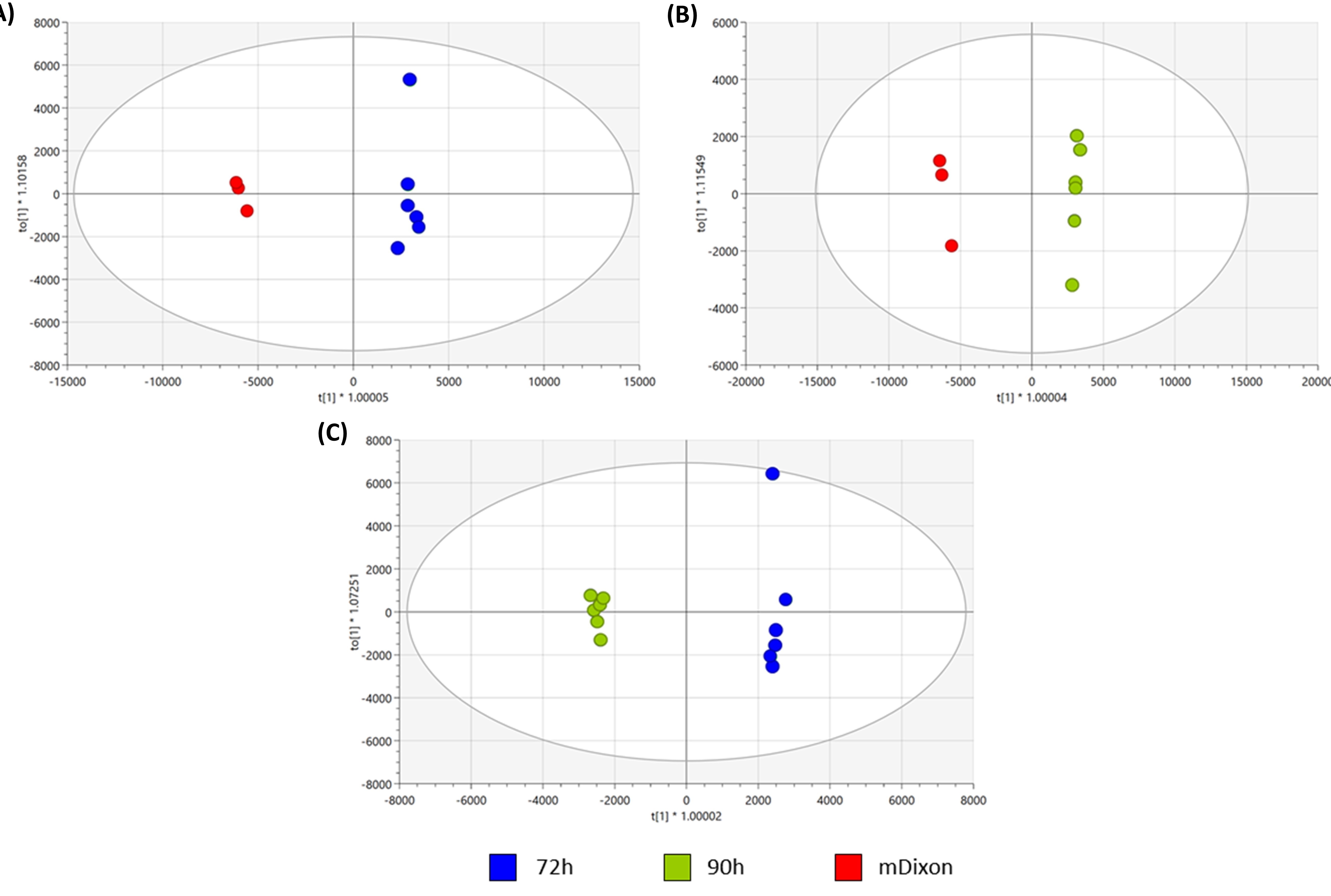

Supplement: Supplemental Material [file KVIR_A_2613494_SM5674.jpg]

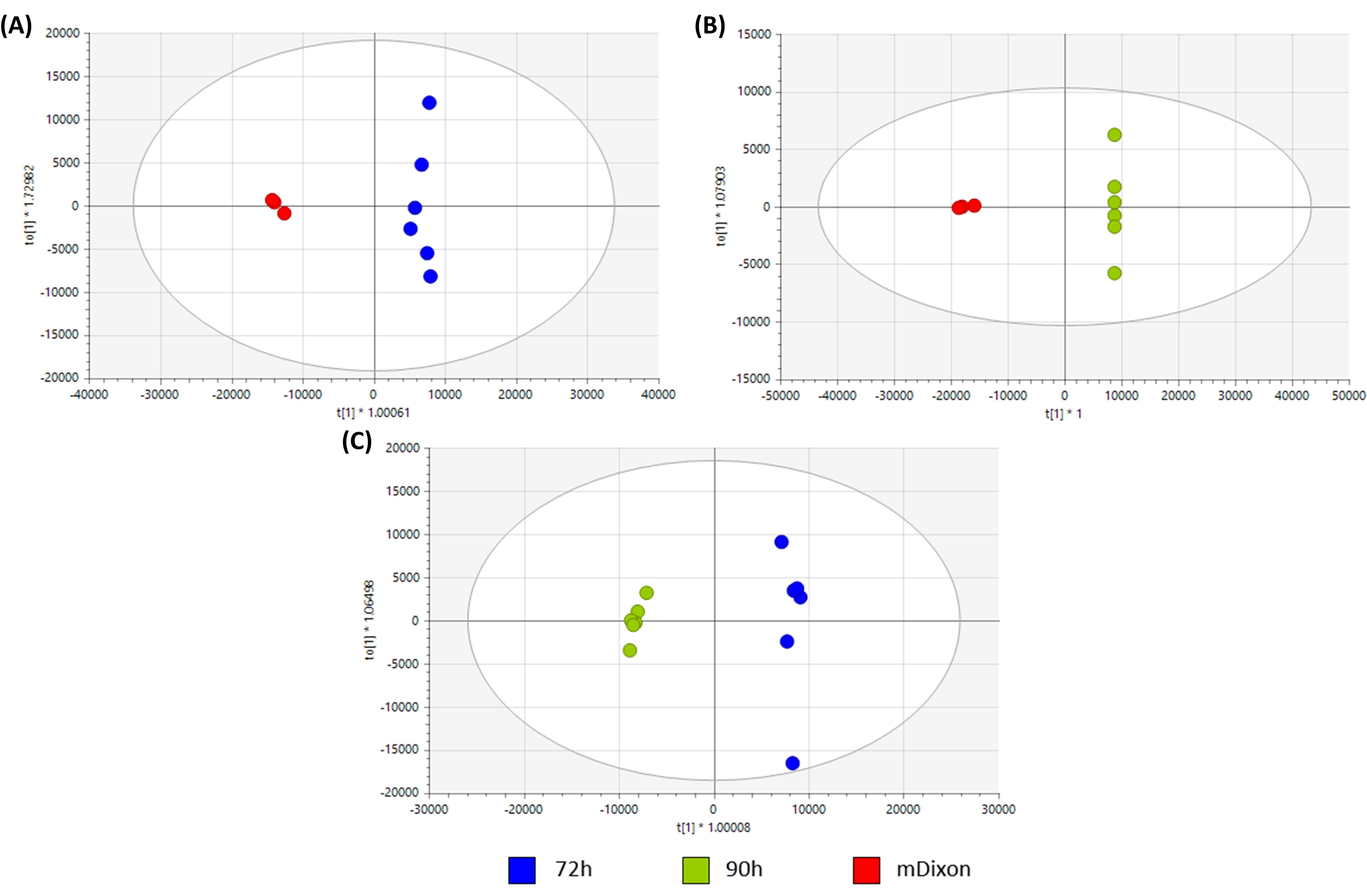

Supplement: Supplemental Material [file KVIR_A_2613494_SM5671.jpg]

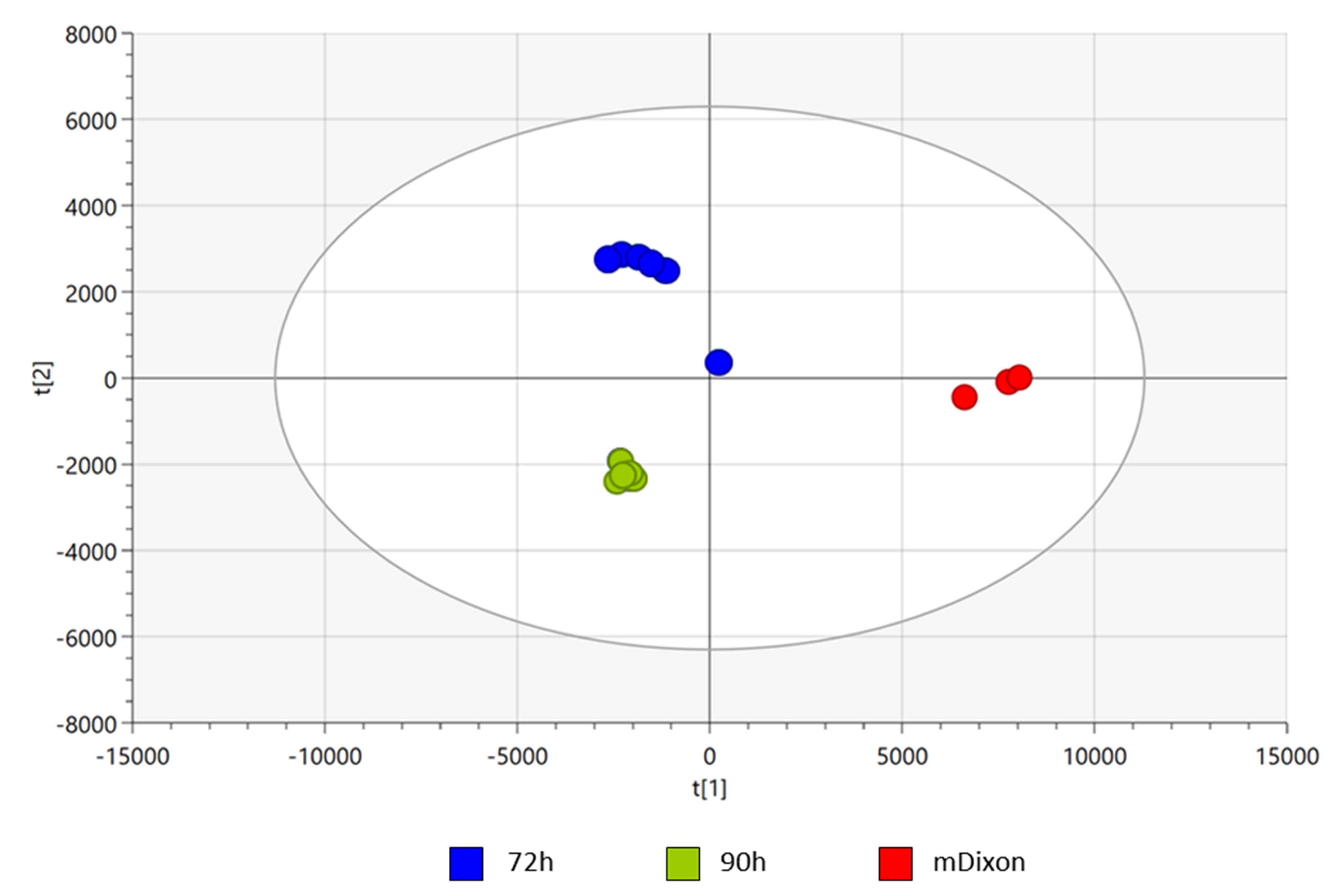

Supplement: Supplemental Material [file KVIR_A_2613494_SM5667.jpg]

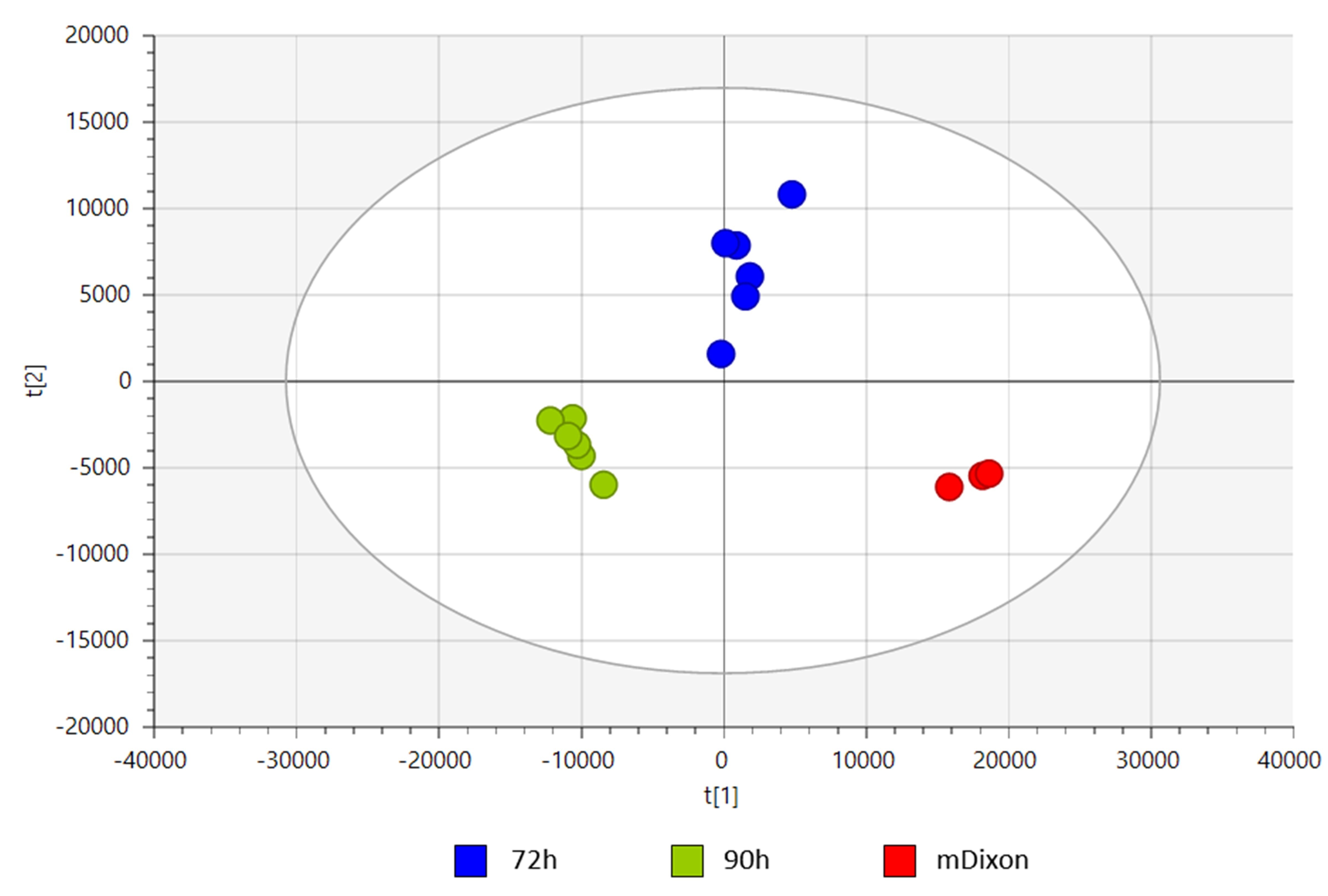

Supplement: Supplemental Material [file KVIR_A_2613494_SM5666.jpg]

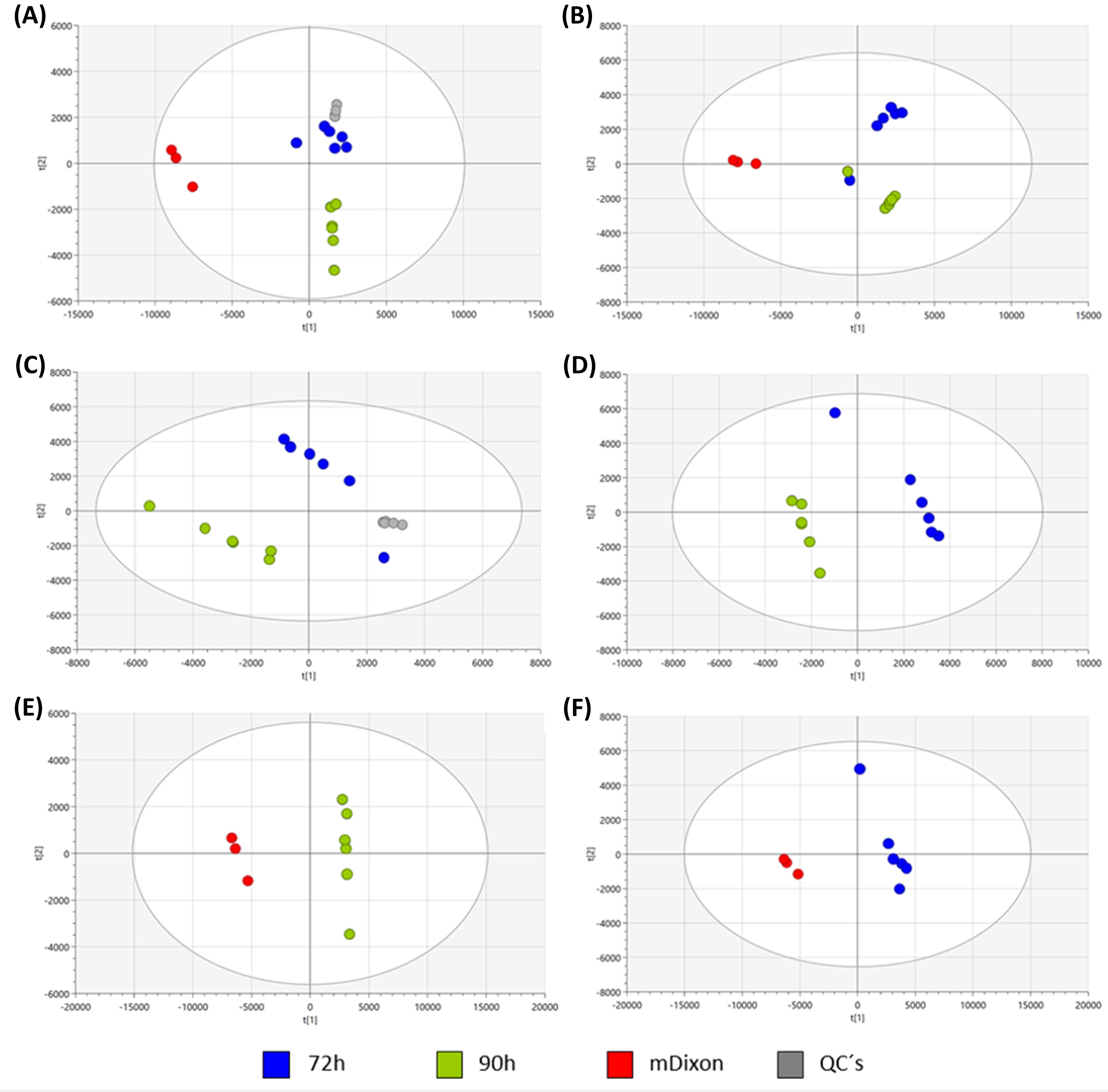

Supplement: Supplemental Material [file KVIR_A_2613494_SM5665.jpg]

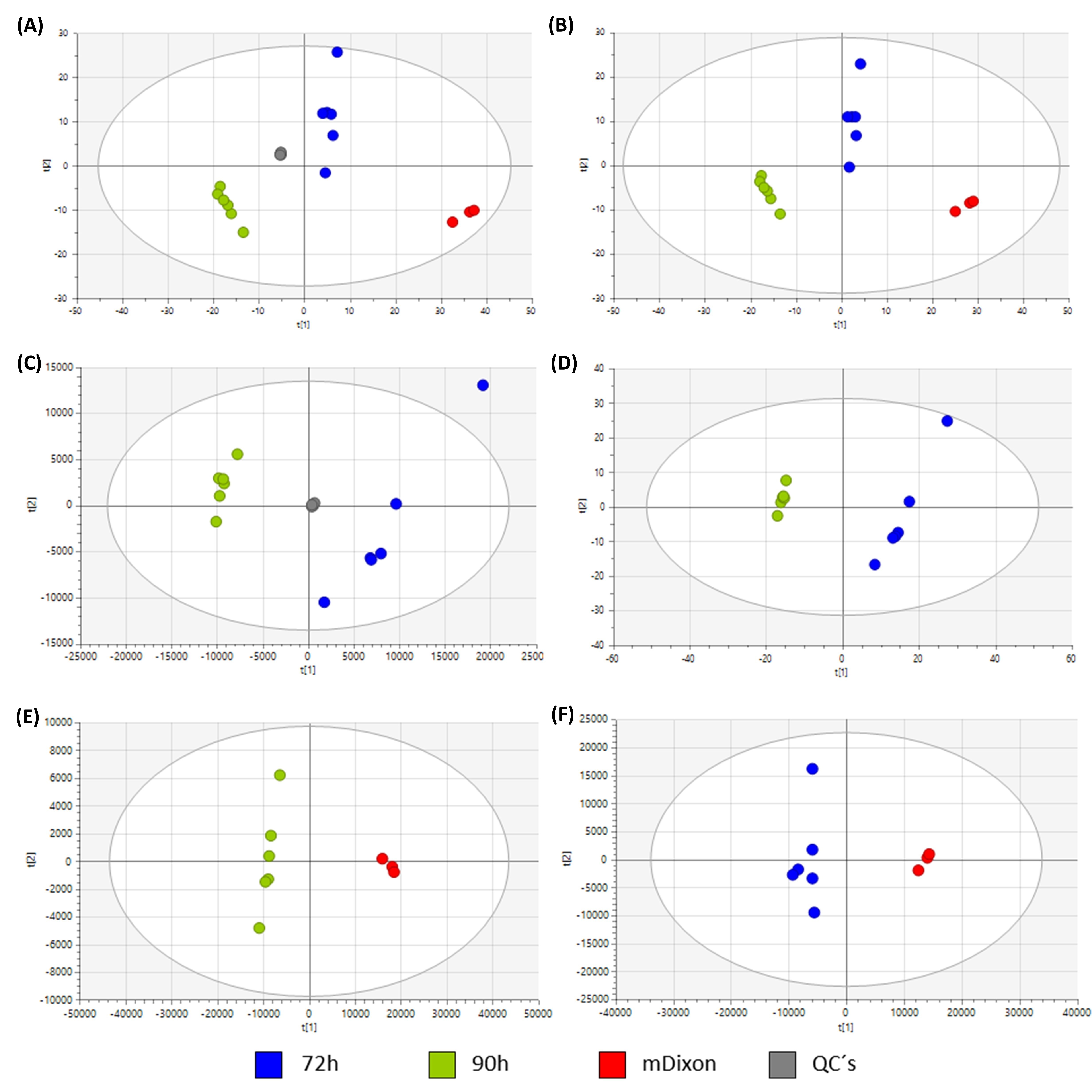

Supplement: Supplemental Material [file KVIR_A_2613494_SM5664.jpg]
