# Supplementary material for: Malassezia globosa lipidome: The dynamics of uptake and secreted lipids
Source: Virulence. 2026 Feb 2;17(1):2613494. doi: 10.1080/21505594.2026.2613494 (PMC12944816; doi:10.1080/21505594.2026.2613494)
Supplement: Supplementary Information.docx [file KVIR_A_2613494_SM8653.docx]

**Supplementary Information**

Supplementary Table 1. Eighty-five different lipids were identified during lipidomic analysis.

| **Lipid Family** | **Lipid Class** | **Lipid Formula** | **Identification Level** |
| --- | --- | --- | --- |
| Fatty Acyls (FA) | Fatty acyl carnitines (CAR) | CAR (20:4) | 3 |
|  | Fatty Acids and Conjugates (FC) | FA (16:0) | 2 |
|  |  | FA (16:1) | 2 |
|  |  | FA (18:0) | 2 |
|  |  | FA (18:0;O) | 3 |
|  |  | FA (18:1) | 2 |
|  |  | FA (18:2) | 2 |
|  |  | FA (20:4) | 3 |
|  | Fatty alcohols (FOH) | FOH (9:1) | 3 |
|  | Fatty acyl homoserine lactones (HSL) | FA (16:1-HSL) | 3 |
|  | Fatty amides (NA) | NA (18:1;O2) | 3 |
|  |  | NA (20:1;O2) | 2 |
|  |  | NA (20:2;O2) | 3 |
| Glycerolipids (GL) | Monoacylglycerols (MG) | MG (18:1) | 3 |
|  | Diacylglycerols (DG) | DG (37:4) | 3 |
|  |  | DG (39:5) | 3 |
|  |  | DG (20:0) | 3 |
|  |  | DG (35:6) | 3 |
|  | Triacylglycerols (TG) | TG (44:2) | 3 |
| Glycerophospholipids (GP)  Glycerophospholipids (GP) | Cardiolipins (CL)  Cardiolipins (CL) | CL (47:2) | 3 |
|  |  | CL (49:2) | 3 |
|  |  | CL (54:4) | 3 |
|  |  | CL (56:4) | 3 |
|  |  | CL (58:2) | 3 |
|  |  | CL (62:2)a | 3 |
|  |  | CL (62:2)b | 3 |
|  |  | CL (68:1) | 3 |
|  |  | CL (68:6) | 3 |
|  |  | CL (70:6) | 3 |
|  |  | CL (72:5)a | 3 |
|  |  | CL (72:5)b | 3 |
|  |  | CL (72:8) | 3 |
|  |  | CL (74:4) | 3 |
|  |  | CL (74:6) | 3 |
|  |  | CL(76:13) | 3 |
|  |  | CL (76:2) | 3 |
|  |  | CL (77:2) | 3 |
|  |  | CL (78:14) | 3 |
|  |  | CL (80:12) | 3 |
|  | Monoacylglycerophosphocholines (LPC) | LPC (16:0) | 3 |
|  |  | LPC (21:1) | 3 |
|  | Diacyglycerophosphocholines (PC) | PC (22:2;O2) | 3 |
| Glycerophospholipids (GP) | Diacyglycerophosphocholines (PC) | PC (24:0) | 3 |
|  |  | PC (24:3;O2) | 3 |
|  |  | PC (26:3;O3) | 3 |
|  |  | PC (30:2) | 3 |
|  |  | PC (30:4) | 3 |
|  |  | PC (32:3) | 3 |
|  |  | PC (32:5) | 3 |
|  |  | PC (34:3) | 3 |
|  |  | PC (35:1) | 3 |
|  |  | PC (40:5) | 3 |
|  |  | PC (40:6) | 3 |
|  |  | PC (41:5) | 3 |
|  | Diacylglycerophosphates (PA) | PA (8:0/13:0) | 3 |
|  | Glycerophosphoglycerols (PG) | PG (40:3) | 3 |
|  |  | PG (O-40:5) | 3 |
|  | Glycerophosphoinositols (PI) | PI (O-42:5) | 3 |
| Sphingolipids (PS) | Ceramides (Cer) | CerP (34:1) | 3 |
|  | Phosphosphingolipids (Cer-PI) | Cer-PI (44:0;O2) | 3 |
|  |  | Cer-PI (46:0;O3) | 3 |
| Sterol Lipids (ST)  Sterol Lipids (ST) | Bile acids (BA)  Bile acids (BA) | ST (24:1;O3;G) | 3 |
|  |  | ST (24:1;O4) | 3 |
|  |  | ST (24:1;O4;G)a | 2 |
|  |  | ST (24:1;O4;G)b | 3 |
|  |  | ST (24:1;O4;G)c | 2 |
|  |  | ST (24:1;O4)a | 2 |
|  |  | ST (24:1;O4)b | 2 |
|  |  | ST (24:1;O4)c | 2 |
|  |  | ST (24:1;O4)d | 2 |
|  |  | ST (24:1;O5)a | 3 |
|  |  | ST (24:1;O5)b | 3 |
|  |  | ST (24:1;O5;G) | 2 |
|  |  | ST (24:1;O5;G)a | 2 |
|  |  | ST (24:1;O5;G)b | 2 |
|  |  | ST (24:2;O3) | 3 |
|  |  | ST (24:2;O4) | 3 |
|  |  | ST (24:2;O4)a | 3 |
|  |  | ST (24:2;O4)b | 3 |
|  |  | ST (24:2;O5) | 3 |
|  |  | ST (24:2;O5)a | 3 |
|  |  | ST (24:2;O5)b | 3 |
|  |  | ST (26:2:O2,G)a | 3 |
|  |  | ST (26:2:O2,G)b | 3 |
|  |  | ST (27:2:O4)85 | 3 |

The annotation level documentation was conducted per the directives provided in prior research [96] (Blaženović et al., 2018). The initial level of annotation, which is the most basic, is achieved through the exact mass match (4). This is followed by the confirmation of the molecular formula (3), the identification of fragment signals that are specific to the compound (2), and finally, the confirmation at the standard level (1).

1. **Supplementary figures**

**
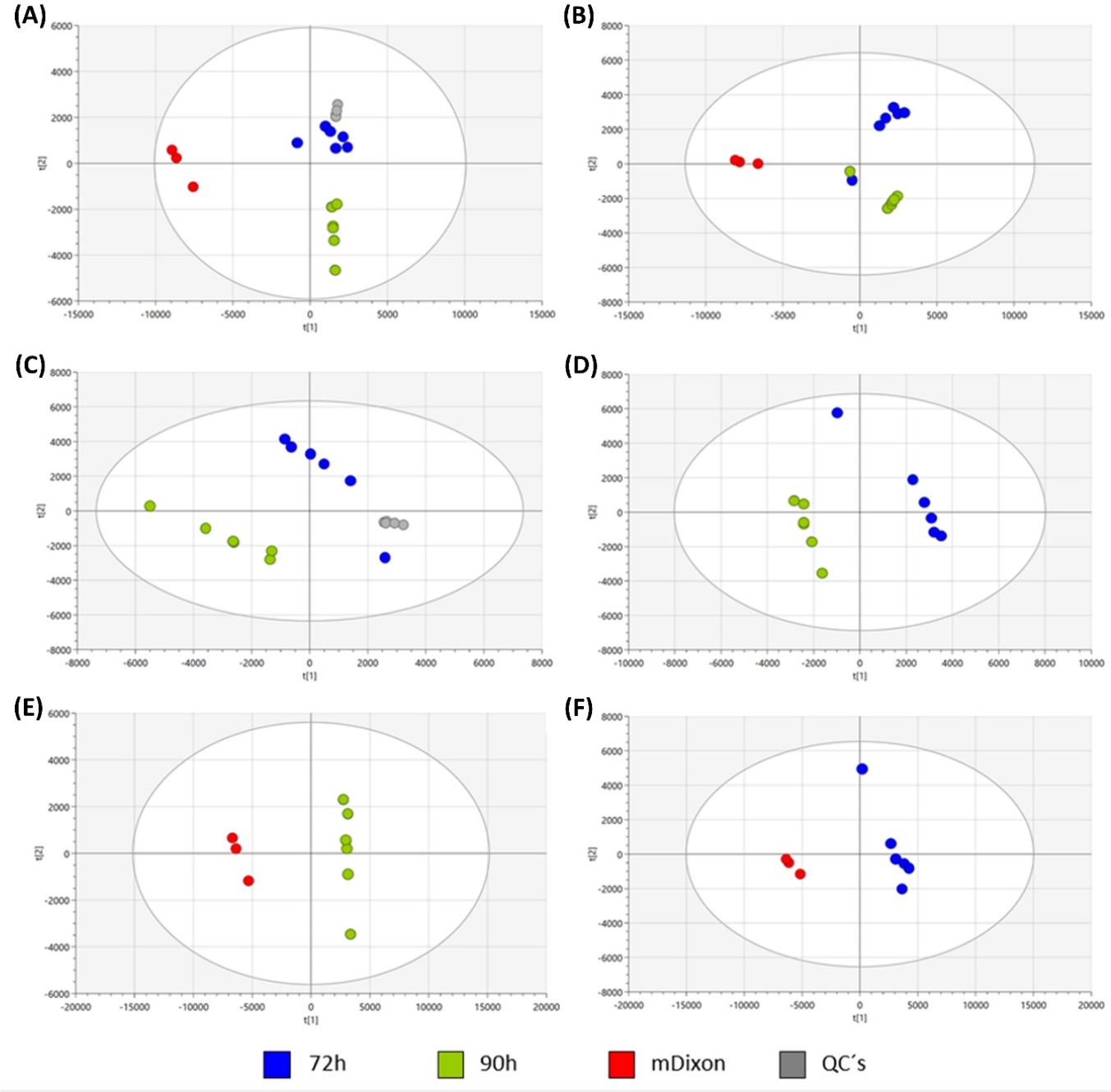
**

**Supplementary figure S1**. PCA-X score plots for negative ionization mode (A) samples, mDixon control and QC´s. R2 = 0.925 Q2 = 0.223 (B) samples and mDixon control. R2 = 0.967 Q2 = 0.92 (C) samples and QC´s. R2 = 0.959 Q2 = 0.847 (D) samples. R2 = 0.98 Q2 = 0.889 (E) 72 h sample and mDixon control. QCs. R2 = 0.983 Q2 = 0.96 (F) 90h sample and mDixon control. QCs. R2 = 0.94 Q2 = 0.864 for negative mode. Red: mDixon control, Green: 90 h sample, Blue: 72 h sample and Gray: QC´s. Ellipse Hotelling´s T2 at α 0.05 is displayed for all graphs.

**Partial Least Squares Discriminant Analysis (PLS-DA)**

**
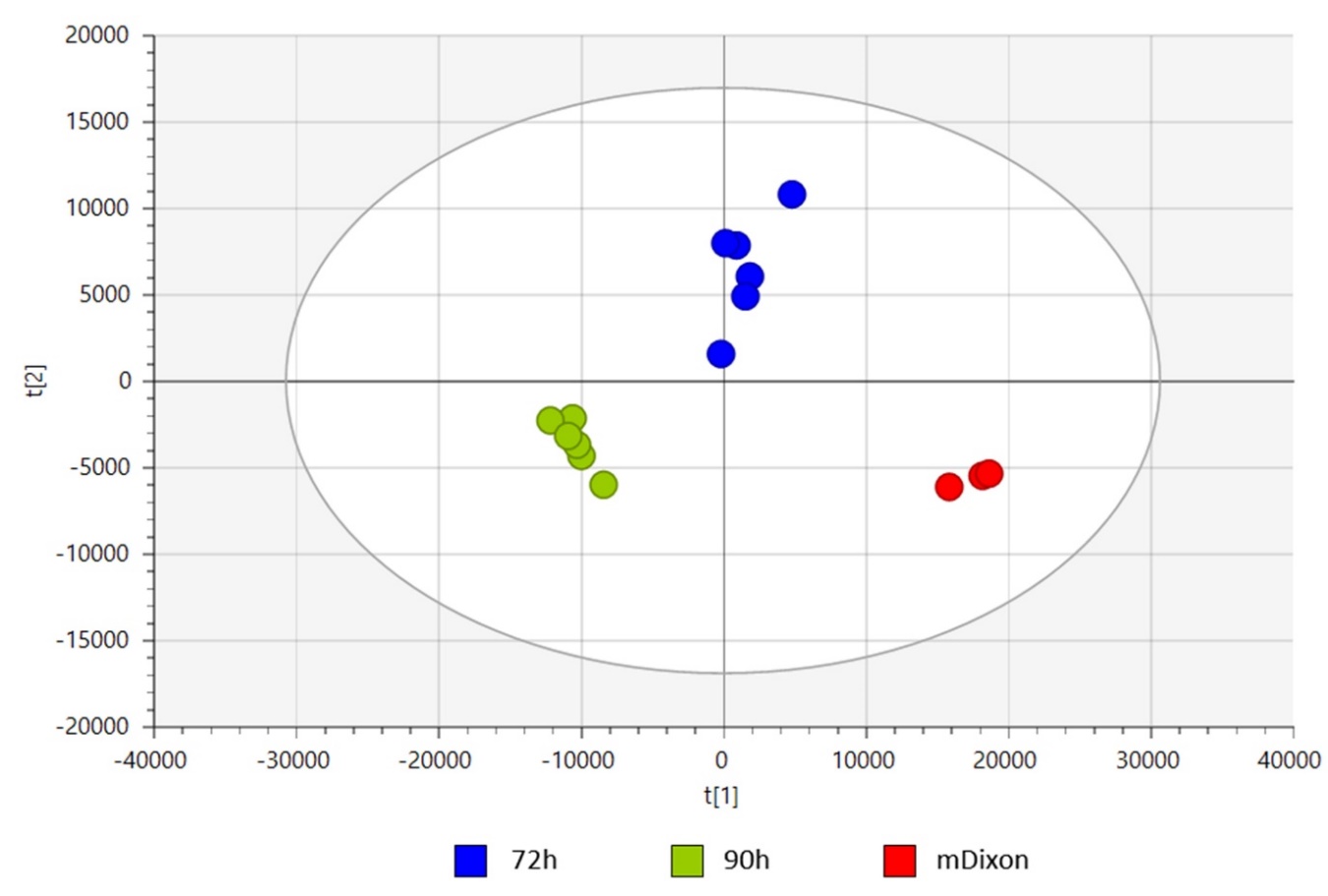
**

**Supplementary figure S2.** PLS-DA score plots for samples in positive ionization mode, mDixon control. R2 = 0.845; Q2 = 0.922; cv-ANOVA: 2.72851e-06. Red: mDixon control, Green: 90 h sample, Blue: 72 h sample. Ellipse Hotelling´s T2 at α 0.05 is displayed for all graphs.


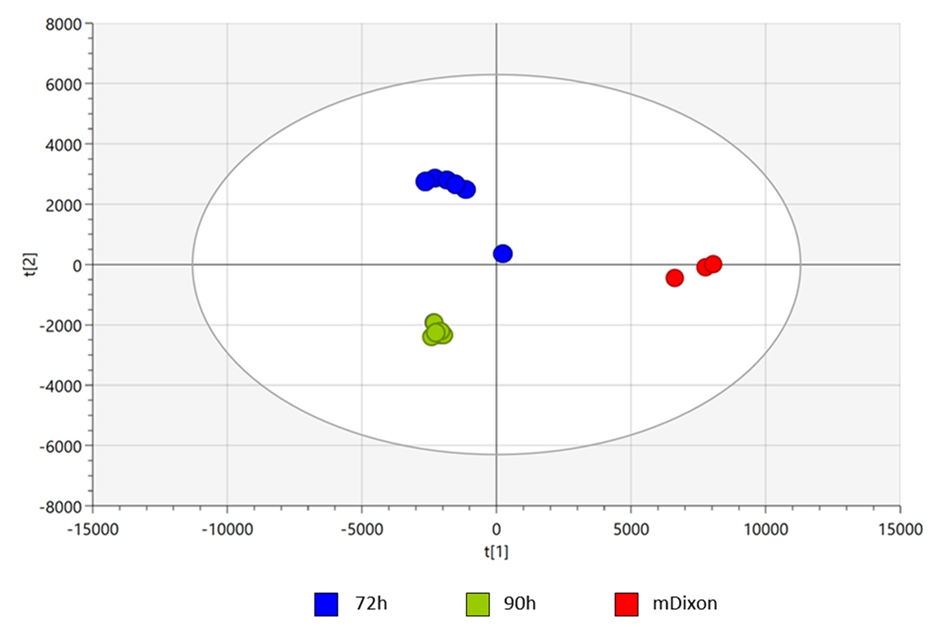


**Supplementary figure S3.** PLS-DA score plots for samples in negative ionization mode. mDixon control. R2 = 0.823; Q2 = 0.912; cv-ANOVA: 4.86369e-10 in negative mode. Red: mDixon control, Green: 90 h sample, Blue: 72 h sample. Ellipse Hotelling´s T2 at α 0.05 is displayed for all graphs.

**Orthogonal Projections to Latent Structures Discriminant Analysis (OPLS-DA)**

**
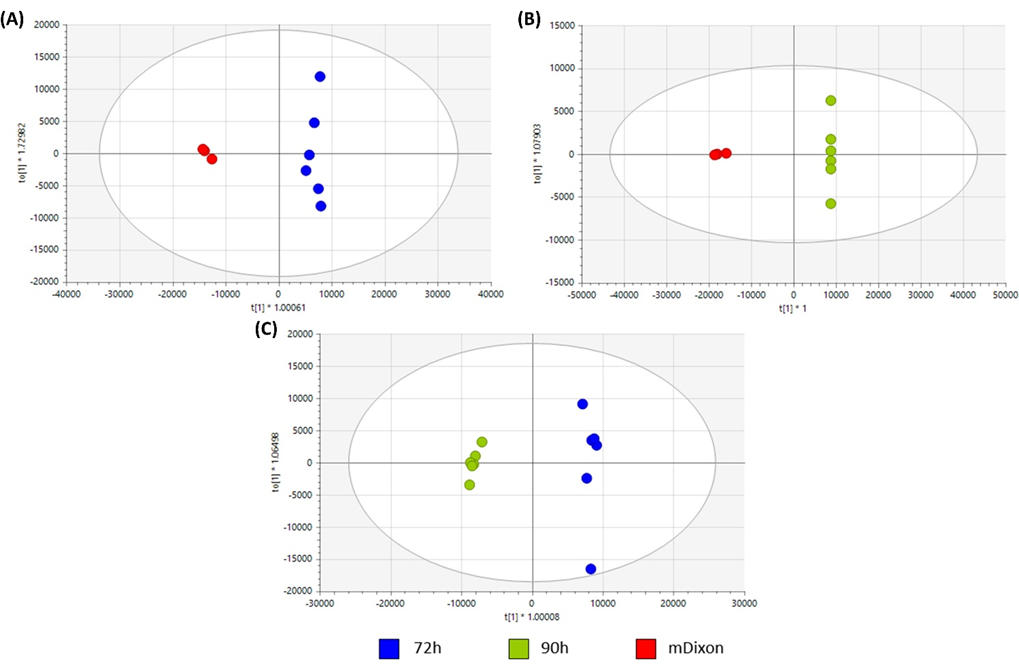
**

**Supplementary figure S4**. OPLS-DA score plots in positive ionization mode. (A) 72h sample and mDixon control. R2 = 0.939; Q2 = 0.95; cv-ANOVA: 0.00087485. (B) 90h sample and mDixon control. R2 = 0.983; Q2 = 0.942; cv-ANOVA: 8.897e-05. (C) 72h sample and 90h sample. R2 = 0.886; Q2 = 0.988; cv-ANOVA: 1.07764e-06 in positive mode. Red: mDixon control, Green: 90 h sample, Blue: 72 h sample. Ellipse Hotelling´s T2 at α 0.05 is displayed for all graphs.


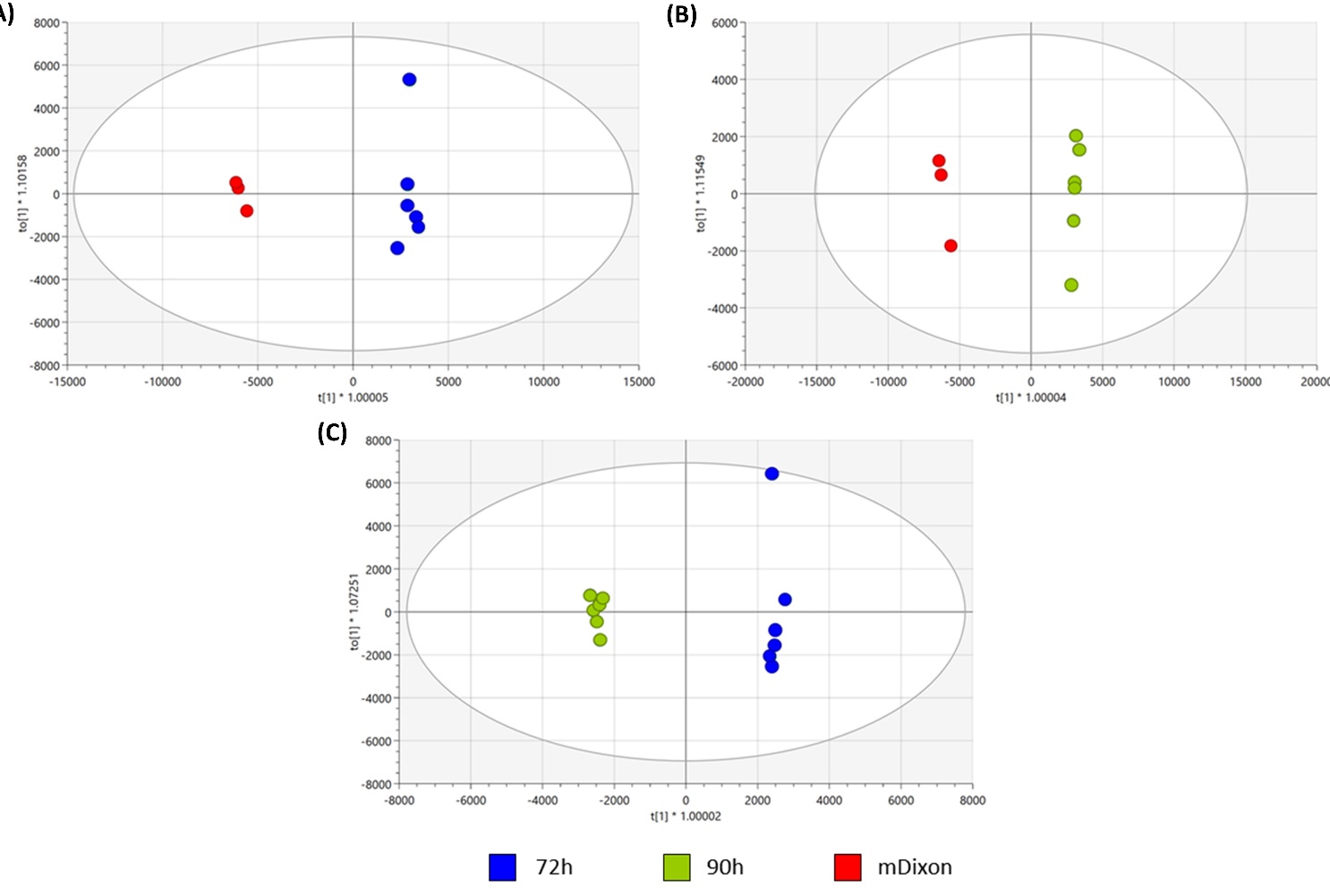

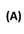


**Supplementary figure S5**. OPLS-DA score plots in negative ionization mode. (A) 72h sample and mDixon control. R2 = 0.94; Q2 = 0.984; cv-ANOVA: 0.000226528. (B) 90h sample and mDixon control. R2 = 0.962; Q2 = 0.991; cv-ANOVA: 0.000746243 (C) 72h sample and 90h sample. R2 = 0.932; Q2 = 0.99; cv-ANOVA: 6.89222e-06 in negative mode. Red: mDixon control, Green: 90 h sample, Blue: 72 h sample. Ellipse Hotelling´s T2 at α 0.05 is displayed for all graphs.

**Principal component analysis (PCA)**

**
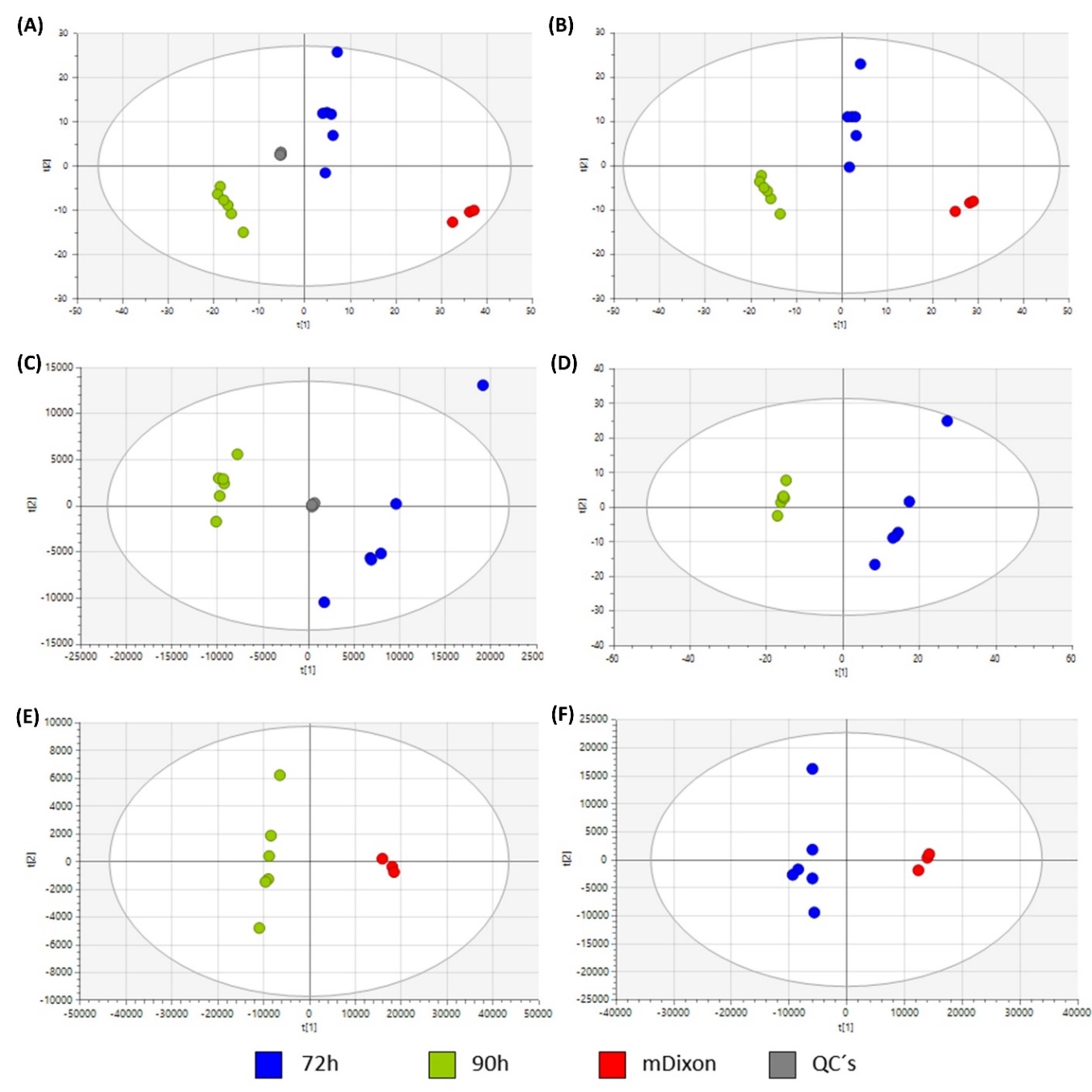
**

**Supplementary figure S6**. PCA-X score plots for positive ionization mode (A) samples, mDixon control and QC´s. R2 = 0.987 Q2 = 0.944 (B) samples and mDixon control. R2 = 0.897 Q2 = 0.914 (C) samples and QC´s. R2 = 0.999 Q2 = 0.962 (D) samples. R2 = 0.993 Q2 = 0.976 (E) 72 h sample and mDixon control. QCs. R2 = 0.988 Q2 = 0.951 (F) 90h sample and mDixon control. QCs. R2 = 0.934 Q2 = 0.912, Red: mDixon control, Green: 90 h sample, Blue: 72 h sample and Gray: QC´s. Ellipse Hotelling´s T2 at α 0.05 is displayed for all graphs.
