# Supplementary material for: Malassezia globosa lipidome: The dynamics of uptake and secreted lipids
Source: Virulence. 2026 Feb 2;17(1):2613494. doi: 10.1080/21505594.2026.2613494 (PMC12944816; doi:10.1080/21505594.2026.2613494)
Supplement: Supplementary Table 1.docx [file KVIR_A_2613494_SM8650.docx]

**Supplementary Table 1.** Eighty-five different lipids were identified during lipidomic analysis.

| **Lipid Family** | **Lipid Class** | **Lipid Formula** | **Identification Level** |
| --- | --- | --- | --- |
| Fatty Acyls (FA) | Fatty acyl carnitines (CAR) | CAR (20:4) | 3 |
|  | Fatty Acids and Conjugates (FC) | FA (16:0) | 2 |
|  |  | FA (16:1) | 2 |
|  |  | FA (18:0) | 2 |
|  |  | FA (18:0;O) | 3 |
|  |  | FA (18:1) | 2 |
|  |  | FA (18:2) | 2 |
|  |  | FA (20:4) | 3 |
|  | Fatty alcohols (FOH) | FOH (9:1) | 3 |
|  | Fatty acyl homoserine lactones (HSL) | FA (16:1-HSL) | 3 |
|  | Fatty amides (NA) | NA (18:1;O2) | 3 |
|  |  | NA (20:1;O2) | 2 |
|  |  | NA (20:2;O2) | 3 |
| Glycerolipids (GL) | Monoacylglycerols (MG) | MG (18:1) | 3 |
|  | Diacylglycerols (DG) | DG (37:4) | 3 |
|  |  | DG (39:5) | 3 |
|  |  | DG (20:0) | 3 |
|  |  | DG (35:6) | 3 |
|  | Triacylglycerols (TG) | TG (44:2) | 3 |
| Glycerophospholipids (GP)  Glycerophospholipids (GP) | Cardiolipins (CL)  Cardiolipins (CL) | CL (47:2) | 3 |
|  |  | CL (49:2) | 3 |
|  |  | CL (54:4) | 3 |
|  |  | CL (56:4) | 3 |
|  |  | CL (58:2) | 3 |
|  |  | CL (62:2)a | 3 |
|  |  | CL (62:2)b | 3 |
|  |  | CL (68:1) | 3 |
|  |  | CL (68:6) | 3 |
|  |  | CL (70:6) | 3 |
|  |  | CL (72:5)a | 3 |
|  |  | CL (72:5)b | 3 |
|  |  | CL (72:8) | 3 |
|  |  | CL (74:4) | 3 |
|  |  | CL (74:6) | 3 |
|  |  | CL(76:13) | 3 |
|  |  | CL (76:2) | 3 |
|  |  | CL (77:2) | 3 |
|  |  | CL (78:14) | 3 |
|  |  | CL (80:12) | 3 |
|  | Monoacylglycerophosphocholines (LPC) | LPC (16:0) | 3 |
|  |  | LPC (21:1) | 3 |
|  | Diacyglycerophosphocholines (PC) | PC (22:2;O2) | 3 |
| Glycerophospholipids (GP) | Diacyglycerophosphocholines (PC) | PC (24:0) | 3 |
|  |  | PC (24:3;O2) | 3 |
|  |  | PC (26:3;O3) | 3 |
|  |  | PC (30:2) | 3 |
|  |  | PC (30:4) | 3 |
|  |  | PC (32:3) | 3 |
|  |  | PC (32:5) | 3 |
|  |  | PC (34:3) | 3 |
|  |  | PC (35:1) | 3 |
|  |  | PC (40:5) | 3 |
|  |  | PC (40:6) | 3 |
|  |  | PC (41:5) | 3 |
|  | Diacylglycerophosphates (PA) | PA (8:0/13:0) | 3 |
|  | Glycerophosphoglycerols (PG) | PG (40:3) | 3 |
|  |  | PG (O-40:5) | 3 |
|  | Glycerophosphoinositols (PI) | PI (O-42:5) | 3 |
| Sphingolipids (PS) | Ceramides (Cer) | CerP (34:1) | 3 |
|  | Phosphosphingolipids (Cer-PI) | Cer-PI (44:0;O2) | 3 |
|  |  | Cer-PI (46:0;O3) | 3 |
| Sterol Lipids (ST)  Sterol Lipids (ST) | Bile acids (BA)  Bile acids (BA) | ST (24:1;O3;G) | 3 |
|  |  | ST (24:1;O4) | 3 |
|  |  | ST (24:1;O4;G)a | 2 |
|  |  | ST (24:1;O4;G)b | 3 |
|  |  | ST (24:1;O4;G)c | 2 |
|  |  | ST (24:1;O4)a | 2 |
|  |  | ST (24:1;O4)b | 2 |
|  |  | ST (24:1;O4)c | 2 |
|  |  | ST (24:1;O4)d | 2 |
|  |  | ST (24:1;O5)a | 3 |
|  |  | ST (24:1;O5)b | 3 |
|  |  | ST (24:1;O5;G) | 2 |
|  |  | ST (24:1;O5;G)a | 2 |
|  |  | ST (24:1;O5;G)b | 2 |
|  |  | ST (24:2;O3) | 3 |
|  |  | ST (24:2;O4) | 3 |
|  |  | ST (24:2;O4)a | 3 |
|  |  | ST (24:2;O4)b | 3 |
|  |  | ST (24:2;O5) | 3 |
|  |  | ST (24:2;O5)a | 3 |
|  |  | ST (24:2;O5)b | 3 |
|  |  | ST (26:2:O2,G)a | 3 |
|  |  | ST (26:2:O2,G)b | 3 |
|  |  | ST (27:2:O4)85 | 3 |

The annotation level documentation was conducted per the directives provided in prior research (Blaženović et al., 2018). The initial level of annotation, which is the most basic, is achieved through the exact mass match (4). This is followed by the confirmation of the molecular formula (3), the identification of fragment signals that are specific to the compound (2), and finally, the confirmation at the standard level (1).
